# Cl-amidine confers organ protection and improves survival in hemorrhagic shock rats via the PAD4-CitH3-NETs axis

**DOI:** 10.1371/journal.pone.0327085

**Published:** 2025-07-01

**Authors:** Huiting Yun, Yunfei Chi, Bin Wei, Hailiang Bai, Weihua Cao, Zheng Zhang, Yufang Zhang, Quanxi Zhang, Hongjie Duan

**Affiliations:** 1 Department of Diagnosis and Treatment for Cadre, The Fourth Medical Center of PLA General Hospital, Beijing, China; 2 Hebei North University, Hebei Province, China; 3 Department of Burns and Plastic Surgery, The Fourth Medical Center of PLA General Hospital, Beijing, China; 4 Research Center of Plastic Surgery Hospital, Chinese Academy of Medical Sciences and Peking Union Medical College, Beijing, China; Versiti Blood Research Institute, UNITED STATES OF AMERICA

## Abstract

**Background:**

The occurrence of multi-organ dysfunction following hemorrhagic shock (HS) remains a critical clinical challenge. The excessive formation of neutrophil extracellular trap (NET) Has been identified as a pivotal pathogenic mechanism. This study preliminarily elucidated the protective mechanism of the PAD4 inhibitor Cl-amidine in a rat model of HS.

**Methods:**

Male Sprague-Dawley rats were subjected to sublethal (40% blood loss, n = 8) or lethal (50% blood loss, n = 10) HS. Rats were divided into Sham group (catheter placement only), HS group (catheter placement followed by blood withdrawal), Vehicle group (0.9% saline), and Cl-amidine (10 mg/kg in 0.9% saline) groups.

**Results:**

Cl-amidine significantly improved the 72-h survival rate and delayed mortality in lethal HS. In Sublethal HS, the drug corrected metabolic disturbances, such as reduced lactate accumulation, while maintaining mean arterial pressure. Mechanistically, the effects of Cl-amidine included reducing circulating cell-free DNA (cf-DNA) and tissue citrullinated histone H3 (CitH3) levels, suppressing PAD4 expression, and improving histopathological outcomes (reduced edema and restored intestinal barrier integrity by upregulation of tight junction proteins Claudin-1/ZO-1). Moreover, Cl-amidine inhibited neutrophil infiltration through ICAM-1 downregulation and reduced the production of TNF-α and IL-6.

**Conclusions:**

In conclusion, Cl-amidine protects against HS by targeting the PAD4-CitH3-NETs axis, breaking the vicious cycle of “NETs-inflammation”, restoring barrier integrity, and alleviating multi-organ damage. The synergistic downregulation of ICAM-1 further enhances the therapeutic efficacy, highlighting Cl-amidine as a novel NETs-modulating strategy for HS. This study provides a theoretical and therapeutic foundation for the prevention and treatment of multi-organ injury following HS.

## 1. Introduction

Hemorrhagic shock (HS), stands as the second leading cause of trauma-related deaths, accounting for 30–40% of global trauma fatalities, with over 50% of these deaths, occurring within 24 hours of injury [1]. While fluid resuscitation is essential for restoring circulation, it paradoxically exacerbates intestinal ischemia-reperfusion injury, triggering a “second hit” characterized by systemic inflammatory response syndrome (SIRS) and multi-organ dysfunction syndrome (MODS) gradually ensue, highlighting the limitations of current strategies that fail to address the inflammatory cascade driving organ failure [2, 3, 4]. During acute blood loss, intestinal ischemia drives mitochondrial dysfunction and necrotic cell death, releasing damage-associated molecular patterns (DAMPs) like HMGB1 and DNA fragments [4,5]. These DAMPs activate the innate immune via system through the TLR4/MyD88 pathway, recruiting neutrophils to the mesenteric microvasculature and disrupting endothelial integrity through glycocalyx shedding, junctional protein degradation, and capillary leakage [6, 7, 8]. Critically, neutrophils amplify injury through neutrophil extracellular traps (NETs) [9,10].

NETs as antimicrobial defense structures composed of DNA scaffolds modified with citrullinated histone H3 (CitH3), myeloperoxidase (MPO), and neutrophil elastase (NE), play a dual role in HS pathophysiology [11, 12, 13]. In HS, excessive NETs impair macrophage polarization toward the anti-inflammatory M2 phenotype, drive pro-inflammatory cytokine (e.g., IL-6, TNF-α) release, and disrupt intestinal tight junctions, exacerbating gut barrier dysfunction [14]. Simultaneously, NETs induce a hypercoagulable state and subsequent hyperfibrinolysis, facilitating microthrombus formation and mucosal bleeding [9,15], positioning them as central mediators bridging localized ischemia to systemic MODS [16, 17, 18].

Peptidylarginine deiminase 4 (PAD4) critically regulates NETs formation by catalyzing the calcium-dependent post-translational conversion of arginine to citrulline residues on histones, a biochemical modification that disrupts histone-DNA electrostatic binding forces and facilitates chromatin decondensation essential for NET formation-driven cell death [19]. Critically, genetic ablation of PAD4 not only abolishes Neutrophil extracellular trap formation (NETosis) but also attenuates tissue damage in ischemia-reperfusion models, establishing its therapeutic potentialt [20,21].

Cl-amidine, a selective PAD4 inhibitor, competitively binds to the enzyme’s active site, inhibiting the production of CitH3 generation and NETosis [22,23]. Preclinical studies have demonstrated its efficacy in treating sepsis by reducing NET-driven coagulation disorders and improving survival rates [24,25], as well as in treating traumatic brain injury by reducing neuroinflammation [26]. However, its potential to improve HS-induced organ damage through modulation of the PAD4/NETs axis has not been explored.

Therefore, this study investigated whether Cl-amidine can alleviate HS-related organ damage by inhibiting PAD4-dependent NETosis, thereby disrupting the inflammatory feedback loop of DAMPs-NETs.

## 2. Materials and methods

### 2.1 Materials and reagents

All experimental materials were procured from certified commercial suppliers. Anesthesia was induced using pentobarbital (Sigma-Aldrich, USA), with tissue fixation performed using 4% paraformaldehyde (Lanjeke Technology, China). The study employed the following primary antibodies: anti-Histone H3 (ab5103; Abcam, UK), anti-Myeloperoxidase (ab208670; Abcam, UK), Neutrophil Elastase antibody (AF0010; Affinity Biosciences, USA), anti-ICAM1 (ab307692; Abcam, UK), anti-Claudin 1 (ab307692; Abcam, UK), ZO-1 antibody (AF5145; Affinity Biosciences, USA) and PADI4 polyclonal antibody (17373–1-AP; Proteintech Group, China). Anti-beta Actin antibody (Rabbit polyclonal, GB15003, Servicebio, China). Corresponding secondary antibodies, including HRP-conjugated and fluorescent dye-labeled (Alexa Fluor 488/594) varieties, were sourced from Servicebio and ZSGB-BIO (China). Molecular biology experiments utilized 2 × SYBR Green Master Mix (G3320-05; Servicebio, China) for quantitative PCR, NovoScript Plus cDNA synthesis kit (E047; GenScript, China) for reverse transcription, and BCA protein assay kit (AR0145; Boster, China) for protein quantification. Surgical procedures were conducted using micro-scissors (G-19745; Heidelberg, Germany) and vascular catheters (Bio-Search Tech, China). DNA extraction was conducted using the QIAamp DNA Mini Kit (Catalog No. 51304; Qiagen, Germany). The CitH3 ELISA kit was purchased from Shanghai Xinweiyu Biotechnology Co., Ltd. (China). TNF-α, IL-10, and IL-6 ELISA kits were obtained from Joyeebio Biotechnology Co., Ltd. (China).

### 2.2 Experimental animals

All experimental protocols strictly complied with the Regulations on the Management of Laboratory Animals (China) and were approved by the Animal Ethics Committee of the Fourth Medical Center of PLA General Hospital (Laboratory Animal License No.: SCXK [Jing] 2024−0001). This study followed the NIH Guidelines for the Care and Use of Laboratory Animals (8th edition, 2011) to ensure ethical rigor and minimize animal suffering. Male Sprague Dawley (SD) rats (body weight: 230–270 g) were purchased from Beijing Keyu Animal Breeding Center. They were housed in the Experimental Animal Center of the Fourth Medical Center of the People’s Liberation Army (PLA) General Hospital under standardized conditions, including ad libitum access to standard pellet feed and drinking water, a 12-h light/dark cycle, controlled temperature (25 ± 2°C), and humidity of 40–60%. The rats were allowed free access to standard pellet feed and drinking water. Before surgery, they were fasted for 12 h with free access to water.

SD rats were anesthetized by intraperitoneal injection of pentobarbital sodium (45 mg/kg). Following anesthesia, After shaving and disinfecting the neck and back regions, the rats were fixed on the operating board, and a 0.5 cm incision was made in the neck to expose the carotid artery and jugular vein. A polyethylene catheter (PU 0.94 × 0.58) was inserted into the right carotid artery for blood withdrawal, sampling, and mean arterial pressure (MAP) monitoring. Another catheter (PU 1.0 × 0.63) was placed in the left jugular vein for drug administration. All catheters were flushed with heparinized saline (100 U/mL) to maintain patency.

Experimental Protocol 1 for Survival Analysis in a Lethal HS Model. Using a random number table, 40 male SD rats were randomly divided into four groups (n = 10 per group). (1) Sham operation group. Rats were anesthetized, followed by arteriovenous catheterization without inducing hemorrhage. (2) HS group. Rats underwent bloodletting through the right carotid artery, withdrawing 50% of their total blood volume, calculated as [body weight (g) × 0.06 + 0.77] × 50% [27,28]. The hemorrhage was performed in two stages, with 25% of blood volume withdrawn over 13 min, followed by an another 25% withdrawn over the next 7 min. Shock induction was confirmed after hemodynamic stability for 30 min. (3) Vehicle group, Rats received 0.5 mL of normal saline through an intravenous catheter after shock induction. (4) Cl-amidine treatment group. Rats were administered 10 mg/kg of Cl-amidine (dissolved in 0.5 mL normal saline) after shock, with dosage and route of administration based on previous research [29]. Following the interventions specific to each group, the rats were housed individually in standardized laboratory cages and continuously monitored for 72 h to evaluate physiological and behavioral responses.

Experimental protocol 2 for survival analysis in a sublethal HS model. Thirty-two rats were randomly divided into four groups (n = 8 per group), with grouping and intervention protocols consistent with those described in Experiment Protocol 1. 40% of the total blood volume (TBV) was withdrawn from the right carotid artery. The TBV was calculated as [body weight (g) × 0.06 + 0.77] × 40%. Blood was withdrawn in two stages, with 20% extracted within the first 7 min and 20% within the following 13 min.

**Fig 1:**
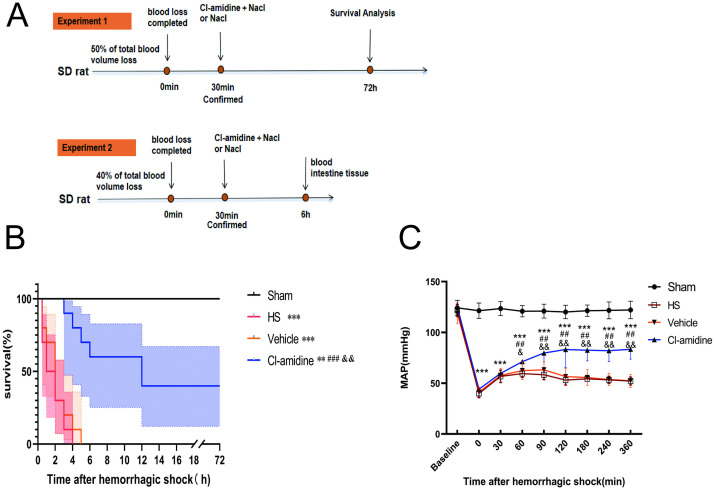
Experimental protocol and therapeutic outcomes of Cl-amidine in hemorrhagic shock. (A) Experimental flowchart, (B) Kaplan-Meier survival curves were generated for all rat groups (n = 10 per group), and intergroup differences were analyzed using the log-rank test, (C) Changes in MAP curves of rats in each group (n = 8), and data are expressed as mean ± SD. Statistical significance was denoted as follows ^**^p < 0.01, ^***^p < 0.001 vs. Sham group; ^*##*^*p < *0.01, ^*###*^*p* < 0.001 vs. HS group; ^*&*^*p < *0.05, ^*&&*^*p < *0.01 vs. Vehicle group.

Six hours after successfully establishing the model, arterial blood was drawn from the carotid artery under anesthesia. The rats were then euthanized, and blood and tissue samples (intestine and lung) were collected and preserved for subsequent analysis. The experimental procedure is illustrated in Fig 1A.

### 2.3 Mean arterial pressure (MAP) measurement

A multichannel electrophysiological system (ADInstruments, Australia) connected to a carotid artery catheter was used to monitor the arterial blood pressure of rats. The Pressure signals were transmitted to a PowerLab data acquisition device (ADInstruments, Australia) connected to a computer. Digitalized hemodynamic data were analyzed using LabChart Pro 8 software (ADInstruments, Australia). For each rat, blood pressure was continuously monitored for 1 minute at each time point, and three random 10-second segments were randomly selected to calculate MAP.

### 2.4 Arterial blood gas analysis

Arterial blood samples were collected via the carotid artery using a arterial blood gas syringe (BD Preset™, Becton Dickinson, USA) and analyzed immediately with a blood gas analyzer (OPTI CCA-TS, USA). The following parameters were measured: Arterial Blood Lactate (LAC), pH, Partial Pressure of Arterial Oxygen (PaO₂), Partial Pressure of Arterial Carbon Dioxide (PaCO₂), Bicarbonate Ion (HCO₃⁻), Base Excess (BE), Oxygen tension at 50% hemoglobin saturation (P50), Arterial Oxygen Saturation (SaO₂).

### 2.5 Serum cell-free DNA (cf-DNA) isolation

Whole blood was centrifuged twice at 12,000 rpm for 10 min at 4°C to separate cells from serum, thereby eliminating interference from white blood cells [30]. According to the manufacturer’s instructions, cfDNA was extracted from 500 μl of serum collected from each rat utilizing the QIAamp DNA Mini Kit (Catalog NO. 51304; Qiagen, Germany). The assessment of cfDNA quality and quantity was performed at the wavelengths of 260 nm and 280 nm employing a NanoDrop micro-spectrophotometer (Thermo Fisher Scientific Inc, Waltham, MA).

### 2.6 Wet/Dry (W/D) weight ratio

The ileum segment of 10 cm was resected at nearly 2 cm from the ileocecal junction and divided into three regions along the direction of blood flow: proximal segment (0–2 cm), middle segment (3–7 cm), and distal segment (8–10 cm). The proximal segment of the ileum was collected for further analysis. Meanwhile, the entire left lung lobe was surgically removed to ensure structural integrity. The collected tissues were then rinsed with phosphate-buffered saline (PBS), and any excess surface moisture was carefully blotted to determine the “wet” weight. Subsequently, the samples were transferred to a vacuum drying oven and dried at 60°C for 72 h to determine the “dry” weight. The wet-to-dry weight ratio (W/D) was calculated by dividing the wet weight by the dry weight.

### 2.7 ELISA

The levels of inflammatory cytokines in the intestinal tissues were assessed using rat specific ELISA kits of tumor necrosis factor (TNF)-α, interleukin (IL)-6, interleukin (IL)-10 and Serum CitH3 following the instructions outlined by the manufacturers.

### 2.8 Hematoxylin & eosin (H&E) staining and pathological scoring

The intestinal and lung tissues were fixed in 4% paraformaldehyde for 48 hours, followed by dehydration, paraffin embedding, and sectioning into 5 μm thick slices for H&E staining. Intestinal damage was assessed using the *Chiu’s* scoring system: 0 (normal mucosa), 1 (subepithelial space formation and villous vacuolization), 2 (epithelial-lamina propria separation), 3 (extensive detachment), 4 (pathological changes in the lower villous region), and 5 (mucosal ulceration with lamina propria disintegration) [31]. Lung injury was evaluated based on four criteria: alveolar congestion/hemorrhage, airway epithelial injury, neutrophil infiltration, and alveolar wall thickening/hyaline membrane formation. Each criterion was scored on a scale of 0–4 (0: normal; 1: < 25% involvement; 2: 25–50%; 3: 50–75%; 4: > 75%). The total score was derived from the sum of all parameters [32]. A double-blind pathologist analyzed three randomly selected areas of each slide at 200 × magnification and calculated the average scores for intestinal and lung tissues from four rats in each group to ensure statistical validity. All procedures followed standardized protocols for tissue fixation, staining consistency, and blinded evaluation to minimize experimental bias.

### 2.9 Immunofluorescence

The tissue samples were dehydrated using a graded ethanol series, followed by paraffin embedding for solidification. Continuous sections of 5μm thickness were prepared and transferred to slides pretreated with poly-L-lysine. The sections were then fixed in a constant temperature oven at 55°C for 2 hours to enhance tissue adhesion. Subsequent processing steps included dewaxing with xylene three times, rehydration with a graded ethanol series, and antigen retrieval. Blocking solution containing 5% normal goat serum and 0.1% Triton X-100 was applied for 30 minutes at room temperature. In this study, a dual immunofluorescence labeling experiment was designed with two experimental groups. Experimental Group I (NE/MPO) was sequentially incubated with rabbit anti-neutrophil elastase antibody (AF0010, Affinity, diluted 1:100, overnight at 4°C) and mouse anti-myeloperoxidase antibody (EPR20257, Abcam, diluted 1:100, 1 hour at 37°C), visualized by Alexa Fluor 594-conjugated goat anti-rabbit IgG (red, 1:50) and Alexa Fluor 488-conjugated goat anti-mouse IgG (green, 1:50). Group 2 (CitH3/NE) utilized rabbit anti-citrullinated histone H3 (Abcam, ab5103, diluted 1:100, overnight at 4°C) combined with mouse anti-NE (1:100, 1 hour at 37°C), with secondary antibodies identical to Group 1. DAPI was used for nuclear counterstaining, and an anti-fade mounting medium was applied. Full-slide scanning was performed using a Nikon A1R HD25 fully automated fluorescence microscope (200 × magnification). The Aipathwell image analysis system (Servicebio) was utilized to quantitatively determine the proportion of co-localized positive cells and the area of the positive regions.

### 2.10 RNA preparation and real-time quantitative PCR (qRT-PCR)

Total RNA was isolated from intestinal tissues utilizing TriQuick reagent (Solarbio, China) by the manufacturer’s guidelines. A total of 1 µg of RNA was subsequently converted into complementary DNA (cDNA) employing a reverse transcription system as per the manufacturer’s recommendations. The primers utilized in this process were custom-sourced from Genecreate Biotech (Wuhan, China), including PAD4 (Forward (F) *5′-CAGCGGTTATTCCAGCAGTGAG-3′* and Reverse (R) *5′-CAAAGAGCCAATCGGAGAAGAGTC-3′*) and β-actin (Forward (F) *5′-CCCATCTATGAGGGTTACGC-3′* and Reverse (R) *5′-TTTAATGTCACGCACGATTTC-3′*). Subsequently, gene expression analysis was performed using the 2^-ΔΔCt^ quantitative method.

### 2.11 Western blotting

Rat intestinal tissues were subjected to lysis utilizing RIPA buffer supplemented with enzymes and protease inhibitors. The overall protein concentration was determined by employing a BCA assay kit (AR0145, Wuhan, China). Following the addition of 4 × loading buffer, which contained β-mercaptoethanol, the samples were subjected to boiling at 100°C for 5 min to facilitate denaturation in the presence of sodium dodecyl sulfate (SDS). The target protein was subsequently resolved via 10% polyacrylamide gel electrophoresis, after which it was transferred onto a PVDF membrane at a voltage of 25V for a period ranging from 30 to 40 min. To block non-specific binding, the membrane underwent treatment with 5% non-fat milk while being continuously shaken for 2 h, followed by an overnight incubation with the primary antibody at a temperature of 4°C. The following day, the membrane underwent three washes with TBST, after which it was incubated with the secondary antibody on a shaker for one hour, followed by three additional washes with TBST before development. The grayscale values of the protein bands, alongside the internal control β-actin, were analyzed utilizing Image J 1.53e software. The specific primary antibodies included Anti-Histone H3 (citrulline R2 + R8 + R17, ab5103, 1:1000, Abcam), recombinant Anti-Claudin 1 (ab307692, at 1:1000, Abcam), ZO-1 (AF5145, 1:1000, affinity), recombinant Anti-ICAM1 (ab282575, 1:1000, Abcam), PADI4 Polyclonal Antibody (17373–1-AP, 1:1000, Proteintech group), and β-actin(GB15003, 1:2000, Servicebio). For the secondary antibody, HRP-conjugated goat anti-rabbit IgG from Servicebio (GB23303) was employed at a dilution of 1:8000.

### 2.12 Statistical analysis

Charts were generated using GraphPad Prism 9.0, while statistical analysis was performed using SPSS R26.0. One-way analysis of variance (ANOVA) was employed to evaluate differences among groups, followed by the least significant difference (LSD) test for subsequent multiple comparisons. Additionally, survival analysis was conducted using the Kaplan-Meier method. A *p*-value of less than 0.05 was considered statistically significant. Results are presented as mean ± standard deviation (SD).

## 3. Results

### 3.1 Cl-amidine enhanced the survival rates in HS rats

As show in Fig 1B, the Kaplan-Meier survival analysis revealed that the 72-hour survival rate was 0% for both the HS and Vehicle groups (n = 10). In contrast,rats treated with Cl-amidine demonstrated a significant improvement in 72-h survival, reaching 40% (*p* < 0.05), indicating a protective effect of Cl-amidine against HS. Analysis of survival time distribution showed that the median survival time for the HS group was 1 hour, with 70% of deaths occurring within 0.5–2 h after shock induction. The median survival time for the Vehicle group was also 1 h, but its survival curve exhibited a continuous downward trend during the 72-h observation period. Notably, Cl-amidine treatment significantly extended the median survival time to 12 h compared to both the HS and Vehicle groups (*p* > 0.05). No statistical difference was observed between the Vehicle and HS groups (*p* > 0.05).

### 3.2 Cl-amidine increases the MAP in rats with hemorrhagic shock

As showm in Fig 1C, there was no statistically significant difference in baseline MAP between the sham group, HS group, Vehicle group, and Cl-amidine group (121.3 ± 6.8 vs 122.1 ± 7.2 vs 120.8 ± 6.5 vs 121.4 ± 7.6 mmHg, *p* > 0.05). After HS induction, the MAP of the HS, Vehicle, and Cl-amidine groups was significantly lower compared to the Sham group (39.9 ± 4.1, 41.2 ± 4.7, and 44.2 ± 5.9 mmHg vs 121.4 ± 7.6 mmHg, *p* < 0.001), with no intergroup differences observed (*p* > 0.05). At 60 minutes, the MAP of rats treated with Cl-amidine was 71.3 ± 7.0 mmHg, significantly higher than the Vehicle group (62.5 ± 8.7 mmHg; *p* < 0.05). This difference widened at 90 minutes (79.8 ± 16.7 vs 63.2 ± 7.9 mmHg; *p* < 0.05). After 120 minutes, the MAP in the Cl-amidine group remained at 83.3 ± 17.6 mmHg, significantly above the Vehicle level (56.6 ± 8.1 mmHg; *p* < 0.001). After180 minutes, there was a continued improvement in MAP (82.5 ± 10.5 vs 55.6 ± 7.3 mmHg; *p* < 0.01). At 240 minutes, the MAP remained elevated (82.4 ± 10.6 vs 53.6 ± 6.0 mmHg; *p* < 0.01). After 360 minutes, the Cl-amidine group stabilized at 83.4 ± 9.6 mmHg, while the Vehicle group decreased to 52.4 ± 6.4 mmHg (*p* < 0.01). It’s worth noting that rats treated with Cl-amidine maintained a MAP significantly lower than the Sham group level throughout the process (*p* < 0.001), indicating partial, rather than complete, hemodynamic recovery. No statistical difference was observed between the Vehicle group and the HS group at any time point (*p* > 0.05).

### 3.3 Cl-amidine improves arterial blood gas in rats with hemorrhagic shock

**Fig 2:**
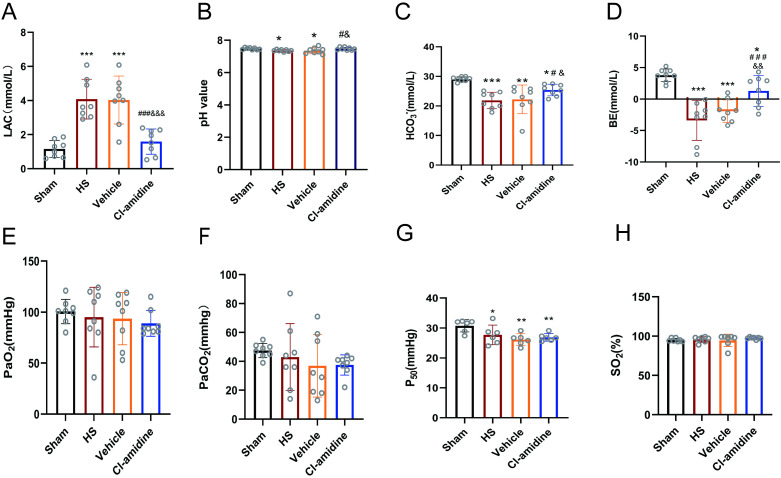
Effect of Cl-amidine on arterial blood gas in rats with hemorrhagic shock. **(A)** Lactate (LAC, n = 8), (B) pH value (pH, n = 8), **(C)** Bicarbonate ion (HCO₃ ⁻ , n = 7), **(D)** Base excess (BE, n = 8), **(E)** Partial pressure of arterial oxygen (PaO₂, n = 8), **(F)** Partial pressure of arterial carbon dioxide (PaCO₂, n = 8), **(G)** P50 oxygen tension (n = 6), **(H)** Arterial oxygen saturation (SaO₂, n = 8). Data are expressed as mean ± SD, Statistical significance was denoted as follows ^*^p < 0.05, ^**^p < 0.01, ^***^p < 0.001 vs Sham; ^#^*p* < 0.05, ^###^*p* < 0.001 vs HS; ^&^*p* < 0.05, ^&&^*p* < 0.01, ^&&&^*p *< 0.001 vs Vehicle.

As shown in Fig 2, after 6 h of HS, the LAC level was increased significantly compared to both HS and Vehicle groups (*p* < 0.001). Conversely, there was a notable decrease in pH (*p* < 0.05 compared to both HS and Vehicle), HCO_3_^-^ (*p* < 0.001 compared to HS and *p* < 0.01 compared to Vehicle), and BE (*p* < 0.001 compared to both HS and Vehicle), indicating that HS induced metabolic acidosis. The average pH values recorded for the HS and Vehicle groups were 7.36 ± 0.55 mmol/L and 7.35 ± 0.18 mmol/L, respectively. Although these values were lower than that observed in the sham group (*p* < 0.05), they still fell within the normal physiological range. Therefore, the rats exhibited a state of compensatory metabolic acidosis after enduring 6 h of shock. There are no statistically significant differences were identified in PCO_2_ and PO_2_ among those groups (*p* > 0.05).

However, treatment with Cl-amidine led to significant improvements in the aforementioned parameters, including LAC (*p* < 0.001 compared to both HS and Vehicle), pH (*p* < 0.05 compared to both HS and Vehicle), HCO_3_^-^ (*p *< 0.05 compared to both HS and Vehicle), and BE (*p* < 0.001 compared to HS, *p* < 0.01 compared to Vehicle).The P_50_ value was significantly decreased after HS compared to sham group(*p* < 0.01), indicating a left shift in the oxygen dissociation curve, which suggested an increased affinity of hemoglobin for oxygen, pointing to tissue hypoxia. Additionally, Cl-amidine treatment did not a significant enhancement in P_50_ (*p* < 0.05 compared to HS, *p* > 0.05 compared to Vehicle). There were no statistically significant differences in SO_2_ among the groups (*p* > 0.05 compared to both HS and Vehicle). No statistical differences were observed in the above indexes between the Vehicle and HS groups at any time point (*p* > 0.05).

### 3.4 Cl-Amidine attenuates intestinal and lung tissue damage in hemorrhagic shock

**Fig 3:**
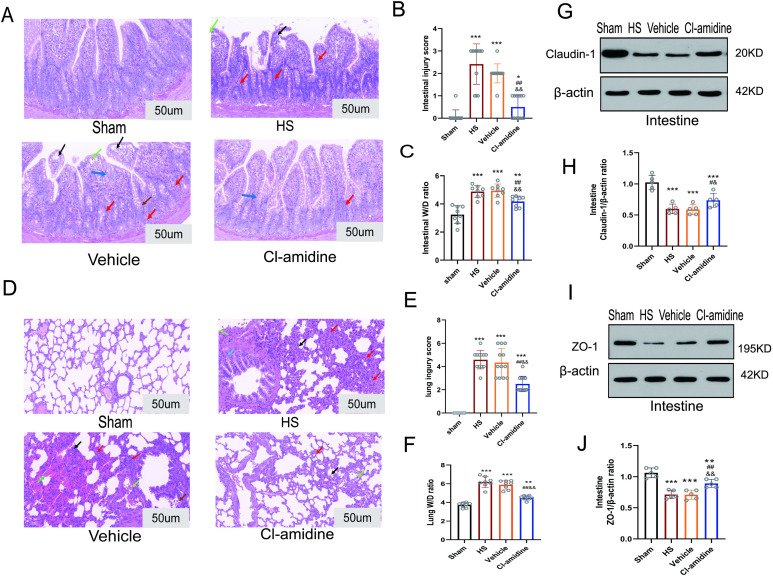
Effect of Cl-amidine on intestinal and lung tissue injury in rats with hemorrhagic shock. **(A)** H&E-stained ileal sections (n = 4, scale bar: 50 µm): Black arrows: Separation of the epithelial apex from the lamina propria; Blue arrows: Swelling of villous epithelial cells; Red arrows: Focal neutrophil infiltration; Brown arrows: Nuclear fragmentation of epithelial cells; Cyan arrows: Lamina propria detachment. **(B)** Pathological injury scores of intestine (n = 4). **(C)** Wet/dry weight ratio of ileal tissues (n = 8). **(D)** H&E-stained pulmonary sections (n = 4, scale bar: 50 µm): Blue arrows: Widened alveolar septa with interstitial edema; Red arrows: Focal neutrophil infiltration; Black arrows: Thickened and disrupted alveolar walls; Brown arrows: Sloughing of pulmonary epithelial cells; Green arrows: Alveolar hemorrhage; Cyan arrows: Leukocyte margination. **(E)** Pathological injury scores of pulmonary tissues (n = 4). **(F)** Wet/dry weight ratio of Lung (n = 8). **(G)** Expression levels of Claudin-1 in intestinal tissue. **(H)** Grayscale value analysis of Claudin-1 expression levels in intestinal tissue. **(I)** Expression levels of ZO-1 in intestinal tissue and corresponding grayscale value analysis. **(J)** Grayscale value analysis of ZO-1 expression levels in intestinal tissue. Data are presented as mean ± SD. Statistical significance was denoted as follows ^*^p < 0.05, ^**^p < 0.01, ^***^p < 0.001 vs. Sham grou*p*; ^#^*p < *0.05, ^##^*p* < 0.01 vs. HS group; ^&^*p < *0.05, ^&&^*p* < 0.01 vs. Vehicle group.

H&E staining demonstrated that Cl-amidine significantly mitigated intestinal and lung tissue damage in HS rats. In Fig 3A-3C, The Sham group exhibited intact intestinal mucosal architecture with orderly arranged villi and tightly connected epithelial-lamina propria junctions, showing no pathological abnormalities. In contrast, both the HS group and Vehicle group displayed severe intestinal injury, characterized by shortened villi, disordered alignment, and epithelial detachment from the lamina propria, accompanied by epithelial cell swelling, increased pathological nuclear division, pyknosis, and karyorrhexis under high magnification. Quantified by the *Chiu’s* pathological scoring system (0–5 points, evaluating villus structure, epithelial integrity, and inflammation), the Sham group scored significantly lower than HS and Vehicle groups (*p* < 0.001). The Cl-amidine-treated grou*p* showed marked improvement, with partial restoration of villus structure, reduced epithelial-lamina propria separation, and diminished nuclear abnormalities, resulting in a significantly lower *Chiu’s* score compared to the Vehicle group (*p* < 0.01). Moreover, the intestinal W/D ratio in the HS and Vehicle grou*p*s was significantly elevated than the Sham group (*p* < 0.001), whereas Cl-amidine significantly reduced the ratio by 14.4% than the Vehicle grou*p* (*p* < 0.01), indicating alleviated edema. No statistical differences were observed between the Vehicle and HS grou*p*s.

In Fig 3D-3F, histopathological analysis of lung tissue revealed intact alveolar architecture in the Sham group. Compared to the Sham group, both HS and Vehicle groups exhibited significant pathological alterations, including narrowed and irregularly sized alveoli, thickened alveolar walls with compromised integrity, widened alveolar septa, interstitial edema, and increased neutrophil infiltration. In contrast, the Cl-amidine group demonstrated near-normal alveolar wall thickness, improved structural integrity, and reduced neutrophil infiltration and hemorrhagic edema. The modified Ashcroft score of the Cl-amidine group was significantly lower than those of the HS and Vehicle groups (*p* < 0.01). Quantitative analysis showed that the lung W/D ratio was markedly elevated in the HS and Vehicle groups compared to the Sham group (*p* < 0.001). Cl-amidine significantly reduced the pulmonary W/D ratio by 23.6% than the Vehicle group (*p* < 0.01). No statistically significant differences were observed between the Vehicle and HS groups (*p* > 0.05).

Tight junction proteins (TJPs, ZO-1 and Claudin-1) are key components of intercellular junctions, playing an important role in maintaining and regulating the constructive integrity of epithelial cell connections [33]. As shown in Fig 3G-3J, at 6 h after HS, the protein expression levels of Claudin-1 and ZO-1 in the HS and Vehicle groups were significantly lower than the sham group. (*p* < 0.001). In contrast, the protein levels of Claudin-1 and ZO-1 in the Cl-amidine group were significantly increased than the HS and Vehicle groups (*p* < 0.05, *p* < 0.01).

### 3.5 Cl-amidine reduces neutrophil infiltration and inflammatory cytokine levels

**Fig 4:**
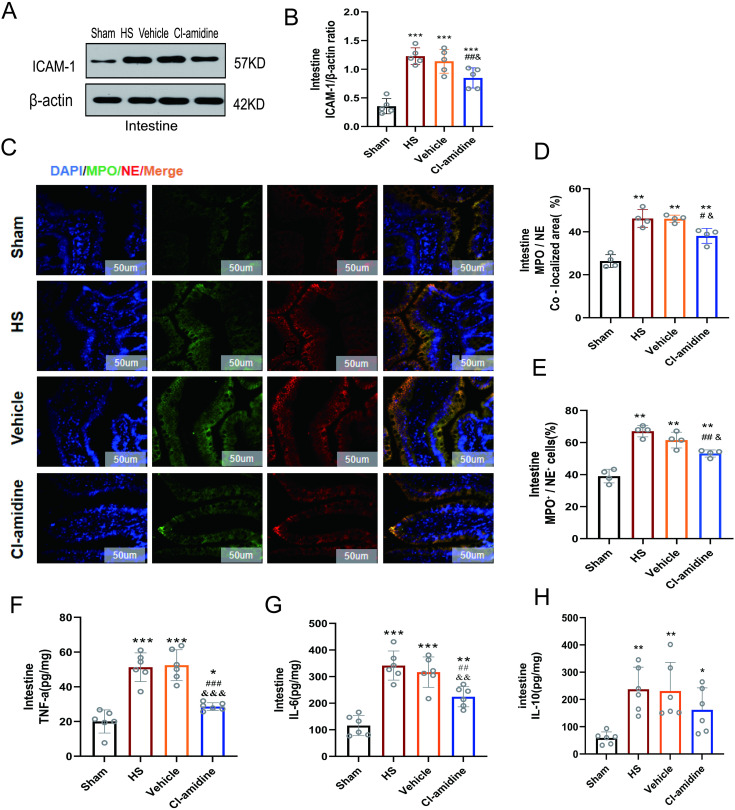
The effects of Cl-amidine on epithelial activation-induced neutrophils and inflammatory responses. **(A)** Expression levels of ICAM-1 in intestinal tissue at 6 hours in different groups (n = 5). **(B)** Expression levels of CitH3 and grayscale value analysis in intestinal tissue at 6 hours in different groups. **(C)** Representative fluorescent staining images of NE and MPO in ileum tissue (n = 4, 200 × , scale bar: 50 µm). Fluorescence channels: NE (red), MPO (green), DAPI (nucleus, blue). **(D)** Percentage of MPO/ NE co-positive area in intestinal tissue. **(E)** Percentage of MPO/ NE co-positive cells in intestinal tissue. **(F)** TNF-α levels in intestinal tissue (n = 6). **(G)** IL-6 levels in intestinal tissue (n = 6). **(H)** IL-10 levels in intestinal tissue (n = 6). Data are presented as mean ± SD. Significant differences: ^*^p < 0.05, ^**^p < 0.01, ^***^p < 0.001 vs. Sham group; ^#^*p* < 0.05, ^##^*p* < 0.01, ^###^*p* < 0.001 vs. HS group; ^&^*p* < 0.05, ^&&^*p* < 0.01, ^&&&^*p* < 0.001 vs. Vehicle group.

As a pivotal adhesion molecule regulating neutrophil transendothelial migration, upregulation of intercellular adhesion molecule 1 (ICAM-1) is closely associated with inflammation-mediated organ injury [34]. In Fig 4A-4B, Western blotting revealed significantly elevated ICAM-1 protein expression in the HS and Vehicle groups compared to the Sham group (*p* < 0.001). Cl-amidine intervention markedly reduced ICAM-1 expression by 41.7% compared to the HS group (*p* < 0.01) and 38.9% compared to the Vehicle group (*p* < 0.05). No statistical difference was observed in the ICAM-1 expression between the Vehicle and HS groups (*p* > 0.05).

MPO and NE are two granular components released during neutrophil activation and key constituents of NETs [35,36], The results in Fig 4C-4E indicate that, significantly increased dual-positive (MPO/NE) cell counts and co-localization areas in the HS group compared to Sham group(*p* < 0.01). These alterations were predominantly localized around blood vessels in the intestinal mucosa lamina propria and epithelial cell junctions. Cl-amidine treatment reduced MPO/NE dual-positive cells compared to the HS and Vehicle groups (*p* < 0.05). Cl-amidine treatment also reduced co-localization areas compared to the HS and Vehicle groups (*p* < 0.05). No statistical differences were observed in the MPO/NE dual-positive cells and co-localization areas between the Vehicle and HS groups (*p* > 0.05).

In Fig 4F-4H, there were a 3.0-fold elevation in intestinal IL-6 and 2.6-fold increase in TNF-α levels in the HS group compared to Sham group at 6 h post-shock (*p* < 0.001). Cl-amidine treatment significantly attenuated systemic inflammation, demonstrating a 31.8% reduction in IL-6 levels versus vehicle controls (*p* < 0.01) and 45.4% decreases in TNF-α concentrations compared to vehicle group (*p* < 0.01), while no significant intergroup difference was observed between vehicle and HS controls (*p* > 0.05). The results demonstrated that at 6 h post-HS, the HS and vehicle group exhibited 3.0-fold (*p* < 0.01) and 2.9-fold (*p* < 0.01) increases in IL-10 levels compared to sham group, respectively. However, Cl-amidine treatment did not induce statistically significant alterations in IL-10 levels relative to either the HS or vehicle groups (*p* > 0.05).

### 3.6 NET levels were decreased by Cl-amidine

**Fig 5:**
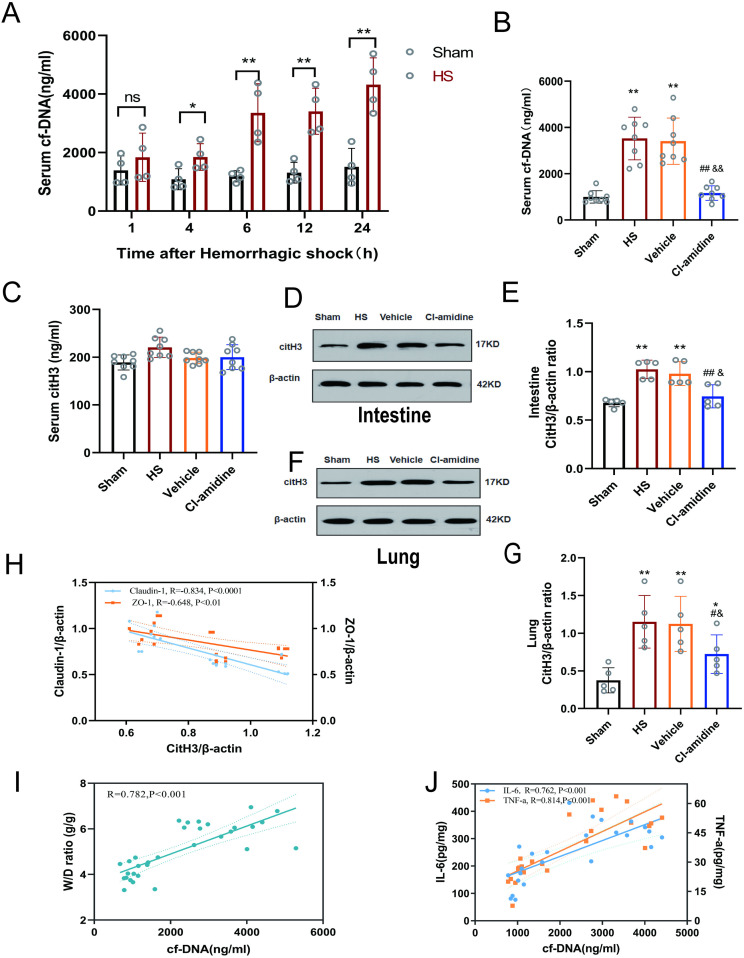
Effect of Cl-amidine on circulating and tissue NETs markers in rats with hemorrhagic shock and correlation analysis between NETs leve is and intestinal tissue injury. **(A)** Changes in serum cf-DNA levels within 24 hours in rats with hemorrhagic shock (n = 4). **(B)** Serum cf-DNA levels at 6 hours in different groups (n = 8). **(C)** Serum CitH3 levels at 6 hours in different groups (n = 8). **(D)** Expression levels of CitH3 in intestinal tissue at 6 hours in different groups (n = 5). **(E)** Grayscale value analysis of CitH3 expression levels in intestinal tissue at 6 hours in different groups. **(F)** Expression levels of CitH3 in lung tissue at 6 hours in different groups (n = 5). **(G)** Grayscale value analysis of CitH3 expression levels in lung tissue at 6 hours in different groups. Data are presented as mean ± SD. Significant differences: * *p* < 0.05, ** *p* < 0.01 vs. sham group; ^#^ *p* < 0.05, ^##^ *p* < 0.01 vs. HS group; ^&^ *p* < 0.05, ^&&^*p* < 0.01 vs. vehicle group. **(H)** Correlation analysis between CitH3 expression levels and Claudin-1/ZO-1 ex*p*ression levels in intestinal tissue using Spearman’s method. **(I)** Correlation analysis between serum cf-DNA and intestinal tissue wet/dry (W/D) weight ratio using Spearman’s method. **(J)** Correlation analysis between serum cf-DNA and intestinal tissue IL-6/TNF-α levels using Spearman’s method.

Cf-DNA, a key biomarker primarily derived from NETs released during cell death, has been demonstrated to predict mortality and complications in critically ill patients, particularly in traumatic settings [37,38]. To evaluate NETs dynamics after HS, serum cf-DNA levels were monitored over 24 h. As shown in Fig 5A-5B, temporal cf-DNA elevation was observed in the HS group compared to the Sham group (1 h: 1.3-fold increase (*p* > 0.05); 4 h: 1.7-fold (*p* < 0.05); 6 h: 2.8-fold (*p* < 0.01);12 h: 2.9-fold (*p* < 0.01); 24 h: 3.0-fold (*p* < 0.01)). A sustained >2-fold elevation from 6 h onward prompted expanded validation at this timepoint. At 6 h, HS group had 3.5-fold higher cf-DNA compared to the Sham group (*p* < 0.01). Cl-amidine efficacy was 67% reduction compared to the HS group (*p* < 0.01) and 66% reduction compared to the Vehicle group (*p* < 0.01). No statistical difference was observed in the cf-DNA level between the Vehicle and HS groups (*p* > 0.05).

CitH3 is a specific and reliable biomarker for NETs [39,40], was quantitatively assessed in this study. In Fig 5C, Serum CitH3 levels showed no statistically significant differences among experimental groups at 6 hours post-HS (*p* > 0.05). As shown in Fig 5D-5G, there was significantly elevated CitH3 expression in the intestinal and lung tissues in the HS and Vehicle groups compared to the Sham group(*p* < 0.01). Cl-amidine treatment significantly reduced CitH3 levels in both intestinal and lung tissues compared to the HS group and Vehicle group (intestinal: *p* < 0.01; lung: *p* < 0.05). No statistically significant difference was observed between the Vehicle and HS groups (*p* > 0.05).

As shown in Fig 5H-5J, the correlation analysis between CitH3 and Claudin-1, ZO-1, cf-DNA and intestinal tissue W/D, as well as cf-DNA and intestinal tissue IL-6, TNF-α was conducted to explore the relationship between NETs levels and tissue injury. High post-shock CitH3 levels showed negative correlations with low Claudin-1 (*r *= −0.834, *p* < 0.001) and ZO-1 (*r* = −0.648, *p* < 0.001). Elevated cf-DNA levels positively correlated with intestinal tissue W/D values (*r* = 0.782, *p* < 0.001) and were also positively associated with intestinal tissue IL-6 (*r* = 0.762, *p* < 0.001) and TNF-α (*r* = 0.814, *p* < 0.001) levels.

### 3.7 Cl-amidine protected intestinal tissues by inhibiting PAD4-induced NETs

**Fig 6:**
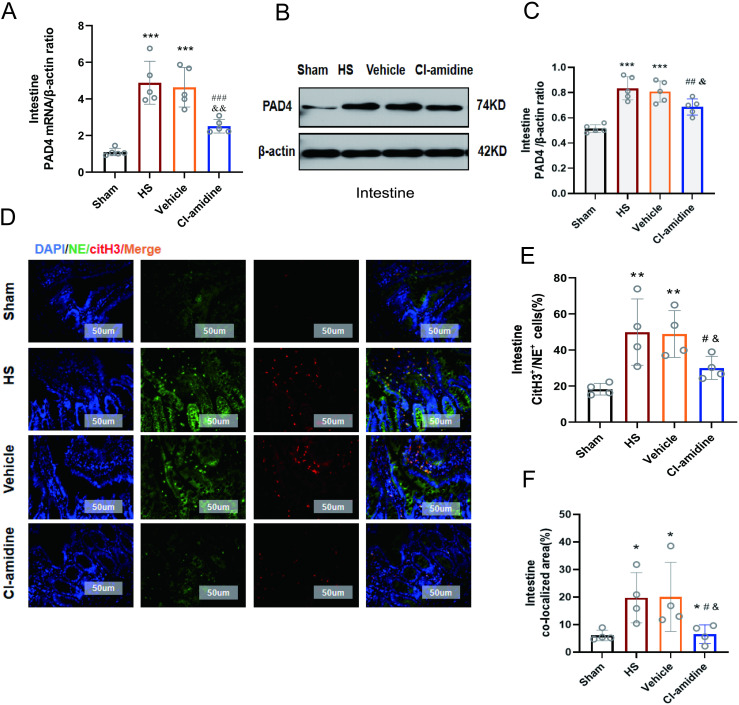
Cl-amidine inhibits PAD4-induced NETs levels. **(A)** Quantitative real-time PCR analysis of PAD4 mRNA expression in intestinal tissue (n = 5). **(B)** Representative Western blot images of PAD4 protein expression in intestinal tissue (n = 5).(**C**) Quantitative analysis of PAD4 protein expression in intestinal tissue. **(D)** Representative immunofluorescence images of CitH3 and NE in intestinal tissue (n = 4, magnification 200 × , scale bar: 50 µm). Fluorescence channels: NE (red), MPO (green), DAPI (nucleus, blue). **(E)** Percentage of CitH3/NE co-positive cells in intestinal tissue. **(F)** Percentage of CitH3/NE co-positive area in intestinal tissue. Data are presented as mean ± SD. Significant differences: ^*^p < 0.05, ^**^p < 0.01, ^***^p < 0.001 vs. Sham group; ^#^*p* < 0.05, ^##^*p* < 0.01 vs. HS group; ^&^*p* < 0.05, ^&&^*p* < 0.01 vs. Vehicle group.

To elucidate the mechanism by which Cl-amidine attenuated intestinal damage, we analyzed PAD4 expression and NETosis. As shown in Fig 6A, PAD4 mRNA level in the HS and Vehicle groups was significantly elevated compared to the Sham group (*p* < 0.001). In contrast, Cl-amidine treatment notably reduced PAD4 mRNA by 48.6% (*p* < 0.001 vs. HS group) and 45.8% (*p* < 0.01 vs. Vehicle group), with no difference between HS and Vehicle groups (*p* > 0.05). In Fig 6B-6C, Western blotting further confirmed that PAD4 protein expression level in the HS and Vehicle groups was markedly increased (*p* < 0.001 vs. Sham), while Cl-amidine significantly suppressed PAD4 protein expression (*p* < 0.01 vs. HS group; *p* < 0.05 vs. Vehicle group).

In Fig 6D-6F, immunofluorescence analysis of CitH3 and NE co-localization demonstrated heightened NET formation in HS and Vehicle groups. Compared to Sham group, the HS group exhibited larger CitH3/NE co-localized areas (*p* < 0.05) and increased co-positive cell ratios (*p* < 0.01). Cl-amidine intervention dramatically reduced both co-localized areas and cell ratios compared to the HS and Vehicle groups (*p* < 0.05).

## 4. Discussion

This study demonstrates that Cl-amidine significantly attenuates HS-induced intestinal and lung injury by inhibiting the PAD4-CitH3-NETs axis, while improving the 72-hour survival rate to 40%. Its protective mechanisms operate at multiple levels. First, Molecular regulation: Cl-amidine directly inhibits PAD4 activity, leading to a 48.6% reduction in intestinal PAD4 mRNA levels and a threefold decrease in CitH3 protein expression, thereby blocking the formation of NETs driven by histone citrullination.

Additionally, Cl-amidine suppresses neutrophil adhesion and infiltration into the intestinal endothelium by downregulating ICAM-1, resulting in reduced release of MPO and NE. Simultaneously, Cl-amidine upregulates tight junction proteins (Claudin-1 and ZO-1), restoring intestinal mucosal barrier integrity and reducing bacterial translocation. These findings align with previous studies indicating the benefits of

PAD4 inhibition in sepsis [24] and ischemia-reperfusion injury [41]. Excessive NETs production is a central factor in intestinal damage following heatstroke, evidenced by a 3.5-fold increase in serum cf-DNA level and a 3.3-fold elevation in intestinal CitH3/NE colocalization at 6 h after HS. Although serum CitH3 levels did not show significant elevation, the marked increase of CitH3 in intestinal and lung tissues (which was suppressible by Cl-amidine) suggests that Shock-induced CitH3 levels may exhibit spatiotemporal heterogeneity in its distribution patterns. This spatial discrepancy could be attributed to preferential activation of tissue-resident neutrophils, delayed entry of CitH3 into circulation due to microvascular barrier functions, or rapid clearance of circulating CitH3 [42,43]. The components of NETs, such as cf-DNA and CitH3 serve as DAMPs, Previous studies have demonstrated that DAMPs can activate the TLR9/MyD88 pathway and its downstream NF-κB signaling, prompting the release of inflammatory cytokines, including IL-6 and TNF-α, from macrophages and endothelial cells [44]. IL-6 and TNF-α are the most common pro-inflammatory cytokines. When IL-6 expression is increased, ileal tight junctions are damaged, which increases the permeability of intestinal wall, leading to bacterial translocation [45]. TNF-α plays an important role in uncontrolled inflammation during ischemia-reperfusion, and affects the expression of tight junction protein ZO-1 in *vitro* experiments [46,47]. Furthermore, our literature review indicates that IL-10 serves as the pivotal anti-inflammatory cytokine within the intestinal immune system. It exerts immunosuppressive effects and downregulates inflammatory responses, thereby representing a critical biomarker of anti-inflammatory regulation in the gut [48], In the current experiment, Cl-amidine treatment did not induce significant alterations in intestinal IL-10 levels, the underlying mechanisms of which require further investigation. This study further confirmes a strong positive correlation between elevated levels of cf-DNA in serum and increased concentrations of TNF-α and IL-6 within tissues. Critically, these cytokines enhance the recruitment of neutrophils, initiating a self-perpetuating cycle: neutrophil infiltration, NETosis, cytokine release, and further neutrophil aggregation. This vicious cycle exacerbates the inflammatory cascade, leading to tissue damage and the deterioration of systemic injury. Previous studies have demonstrated that when phorbol myristate acetate (PMA)-induced NETs are co-cultured with human alveolar epithelial cells and intestinal Caco-2 monolayer epithelial cells, cell death can be triggered within 4 hours [36,41]. These findings further corroborate the pathogenic role of NETs in mediating tissue and organ damage.

Cl-amidine’s cross-organ protection extends to the lungs, reducing pathological injury, edema, and NETs burden, thereby mitigating MODS. This aligns with its efficacy in skin ischemia-reperfusion [49] and brain injury models [50]. Current evidence suggests Cl-amidine may either reinforce endothelial barrier integrity through VE-cadherin upregulation [51] or attenuate microvascular dysfunction via coagulation pathway modulation [25], yet the predominant mechanism remains to be systematically elucidated in disease-relevant models.

There are several limitations in this study. One major constraint is that NETs exhibit a “double-edged sword” role in sterile inflammation. Appropriate levels can clear DAMPs, while excessive release can exacerbates damage [52]. To refine clinical translation strategies, longitudinal monitoring of NETs (e.g., cf-DNA in serum [53], CitH3 [43]) and functional interventions are needed. Another limitation lies in the complexity of in *vivo* systems, which requires validation through conditional PAD4 knockout models or ex vivo intestinal epithelial-NETs coculture system.

## 5. Conclusion

The intestinal ischemia-reperfusion injury induced by HS is driven by excessive neutrophil activation and unconteolled NETosis. The multi-level protection provided by Cl-amidine, a PAD4 inhibitor, including molecular inhibition, tissue repair, and systemic compensation, highlights PAD4 as a promising therapeutic target for shock-related organ damage, highlights PAD4 as a promising therapeutic target for shock-related organ damage. Clinical translation requires further mechanistic dissection and validation of its spatiotemporal efficacy.

## Supporting information

S1 Data Raw date of figures. (XLSX)

S1 File Raw images. (PDF)

## Abbreviations

HS: Hemorrhagic shock
PAD4: Peptidylarginine Deiminase 4
CitH3: HistoneH3 citrullination
NETs: Neutrophil extracellular traps
NETosis: Neutrophil extracellular trap formation
DAMPs: Damage associated molecular patterns
Cf-DNA: Cell free DNA
ICAM-1: Intercellular Adhesion Molecule-1
IL-6: Interleukin 6
TNF-α: Tumor necrosis factor-alpha
LAC: Lactate
MAP: Mean Arterial Pressure
TJPs: Tight Junction Proteins
ZO-1: Zonula Occludens-1.
