# Supplementary material for: Cl-amidine confers organ protection and improves survival in hemorrhagic shock rats via the PAD4-CitH3-NETs axis
Source: PLoS One. 2025 Jul 1;20(7):e0327085. doi: 10.1371/journal.pone.0327085 (PMC12212502; doi:10.1371/journal.pone.0327085)

Intestine Claudin-1(20KDa)

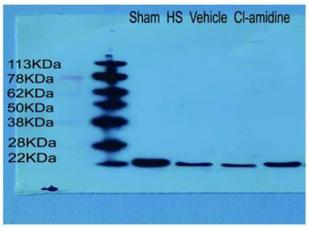

β -actin

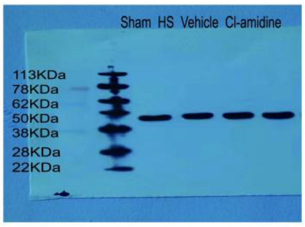

Intestine ZO-1(195KDa)

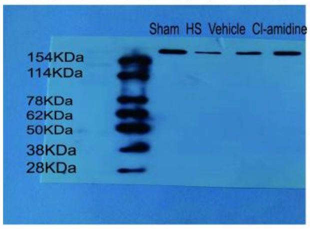

β -actin

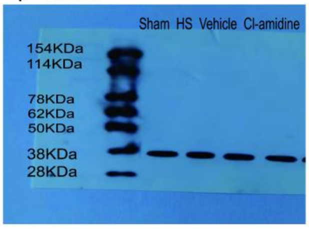

Intestine ICAM-1(57KDa)

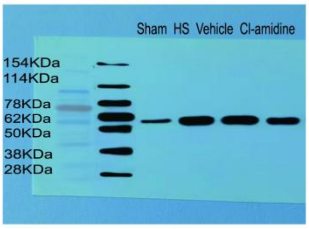

β -actin

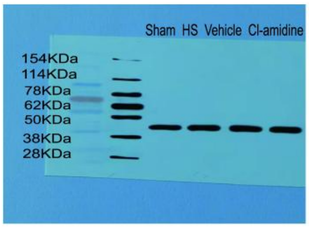

Intestine CitH3(17KDa)

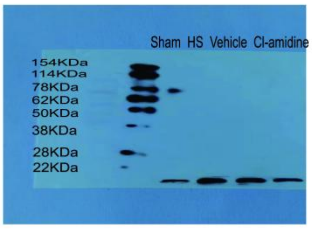

β -actin

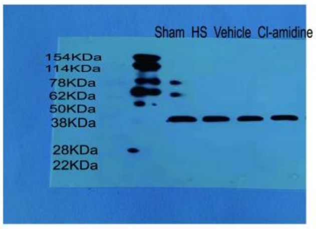

Lung CitH3(17KDa)

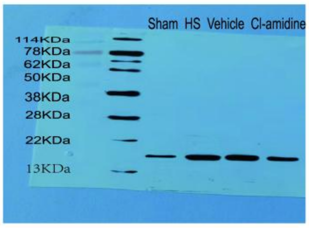

β -actin

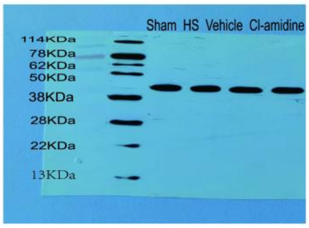

Intestine PAD4(74KDa)

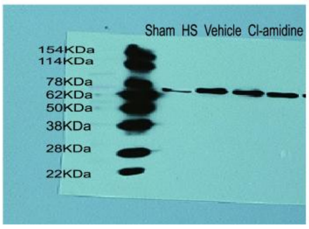

β -actin

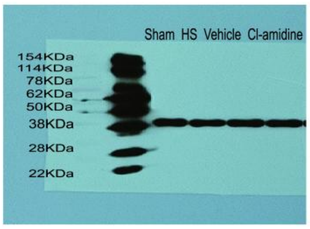

Supplement: S1 File — (PDF) [file pone.0327085.s002.pdf]
